# In vitro monitoring of *HTR2A*-positive neurons derived from human-induced pluripotent stem cells

**DOI:** 10.1038/s41598-021-95041-3

**Published:** 2021-07-29

**Authors:** Kento Nakai, Takahiro Shiga, Rika Yasuhara, Avijite Kumer Sarkar, Yuka Abe, Shiro Nakamura, Yurie Hoashi, Keisuke Kotani, Shoji Tatsumoto, Hiroe Ishikawa, Yasuhiro Go, Tomio Inoue, Kenji Mishima, Wado Akamatsu, Kazuyoshi Baba

**Affiliations:** 1 Department of Prosthodontics, School of Dentistry, Showa University, 2-1-1 Kitasenzoku, Ohta-ku, Tokyo, 145-8515 Japan; 2 Center for Genomic and Regenerative Medicine, School of Medicine, Juntendo University, 2-1-1 Hongo, Bunkyo-ku, 113-8421 Japan; 3 Division of Pathology, Department of Oral Diagnostic Sciences, School of Dentistry, Showa University, 1-5-8 Hatanodai, Shinagawa-ku, Tokyo, 142-8555 Japan; 4 Department of Oral Physiology, School of Dentistry, Showa University, 1-5-8 Hatanodai, Shinagawa-ku, Tokyo, 142-8555 Japan; 5 Exploratory Research Center on Life and Living Systems (ExCELLS), National Institutes of Natural Science, 38 Nishigonaka, Myodaiji, Okazaki-shi, Aichi 444-8585 Japan; 6 Department of System Neuroscience, National Institute for Physiological Science, 38 Nishigonaka, Myodaiji, Okazaki-shi, Aichi 444-8585 Japan; 7 Department of Physiological Science, School of Life Science, SOKENDAI (The Graduate University for Advanced Studies), 38 Nishigonaka, Myodaiji, Okazaki-shi, Aichi 444-8585 Japan

**Keywords:** Neuroscience, Stem cells

## Abstract

The serotonin 5-HT_2A_ receptor (5-HT_2A_R) has been receiving increasing attention because its genetic variants have been associated with a variety of neurological diseases. To elucidate the pathogenesis of the neurological diseases associated with 5-HT_2A_R gene (*HTR2A*) variants, we have previously established a protocol to induce *HTR2A*-expressing neurons from human-induced pluripotent stem cells (hiPSCs). Here, we investigated the maturation stages and electrophysiological properties of *HTR2A*-positive neurons induced from hiPSCs and constructed an *HTR2A* promoter-specific reporter lentivirus to label the neurons. We found that neuronal maturity increased over time and that *HTR2A* expression was induced at the late stage of neuronal maturation. Furthermore, we demonstrated successful labelling of the *HTR2A*-positive neurons, which had fluorescence and generated repetitive action potentials in response to depolarizing currents and an inward current during the application of TCB-2, a selective agonist of 5-HT_2A_Rs, respectively. These results indicated that our in vitro model mimicked the in vivo dynamics of 5-HT_2A_R. Therefore, in vitro monitoring of the function of *HTR2A*-positive neurons induced from hiPSCs could help elucidate the pathophysiological mechanisms of neurological diseases associated with genetic variations of the *HTR2A* gene.

## Introduction

Serotonin is a neurotransmitter involved in many physiological functions in the brain^1,2^. The serotonin 5-HT_2A_ receptor (5-HT_2A_R) is a serotonin receptor subtype encoded by the 5-HT_2A_R gene (*HTR2A*). Mutations in this gene are associated with susceptibility to a variety of neurological diseases. For example, rs6313 (also called T102C^3^) and rs6311, which is in complete linkage disequilibrium with rs6313^4–6^, are genetic polymorphisms of *HTR2A*. They have been associated with schizophrenia^7,8^, psychotic symptoms in Alzheimer’s disease^9^, certain features of depression^10^, sleep breathing disorders^11^, and sleep bruxism^12^.

Understanding the functional consequences of genetic variants is a critical first step towards appreciating their roles in disease development. While previous case–control studies have reported a significant association of genetic polymorphisms of *HTR2A* with a variety of neurological diseases^7–15^, the effect of the variants on disease pathogenesis has not been elucidated in detail, mainly because of the limited accessibility to the brain. In vitro modelling of human diseases using disease-specific human-induced pluripotent stem cells (hiPSCs) has the potential to provide dramatic progress in the elucidation of the pathogenic mechanisms of neurological diseases^16–20^.

To construct an in vitro* HTR2A*-related disease model using hiPSCs, we have previously established a protocol to induce *HTR2A*-expressing neurons from hiPSCs. Here, we investigated the maturation stages and electrophysiological properties of *HTR2A*-positive neurons induced from hiPSCs using our previously established protocol. We also developed reporter lentiviruses, which contain a *HTR2A* promoter-driven fluorescent protein (ZsGreen1) expression construct, to identify and enrich for *HTR2A*-positive neurons. Furthermore, we confirmed the electrophysiological responses of the fluorescently labelled neurons derived from hiPSCs to application of the 5-HT_2A_ R agonist, TCB-2. Our newly established monitoring system of *HTR2A*-positive neurons derived from hiPSCs is expected to be a promising method to elucidate the pathological mechanism of neurological diseases associated with genetic variations of *HTR2A*. To the best of our knowledge, this is the first report of an in vitro, cell-level monitoring system specific to *HTR2A*-positive neurons.

## Results

### Generation and characterization of neurons derived from hiPSCs

**Figure 1:**
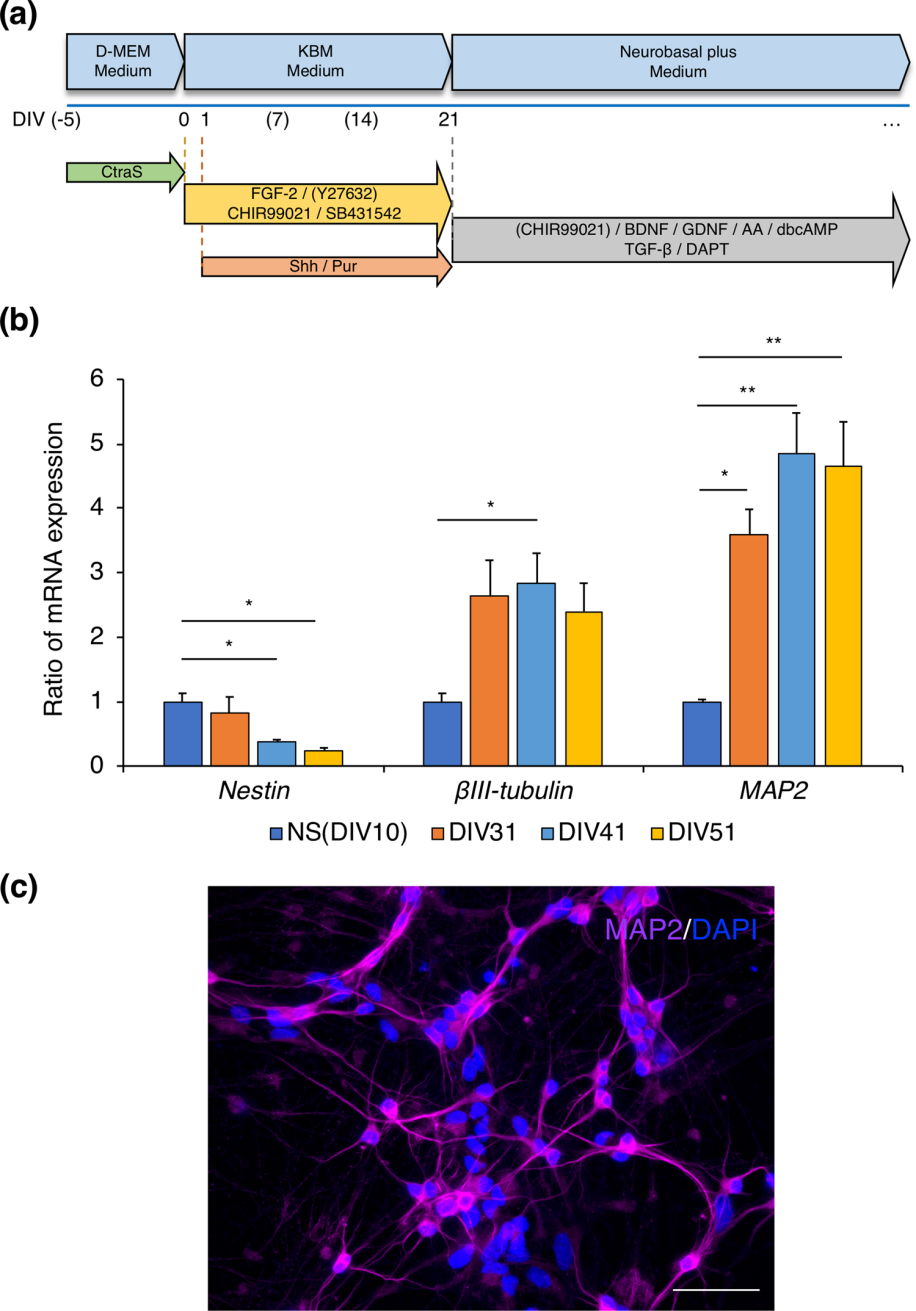
Generation and characterization of neurons induced from hiPSCs. (**a**) A schematic of the protocol for neuronal induction from hiPSCs. The numbers under the line show the days in culture (DIV, days in vitro). (**b**) Gene expression of the neural progenitor cell marker, *Nestin*, and the neuronal markers, *βIII-tubulin* and *MAP2*, in hiPSCs at DIV 10, 31, 41, and 51. The results are expressed as the mean ± s.e.m. of triplicate samples (n = 3). (ANOVA, Dunnet’s test, *p < 0.05, **p < 0.01). (**c**) MAP2 immunofluorescence (magenta) in hiPSCs at DIV 33. A merged image with DAPI-stained nuclei (blue) is shown (scale bar = 50 μm).

The hiPSC line (C2)^20^ was cultured in D-MEM medium to induce a chemically provoked transitional embryoid-body-like state (CtraS)^21^. Neuronal differentiation from hiPSCs was conducted according to a previously reported protocol^20^ with slight modification (Fig. 1a). The day when the medium was changed to KBM medium, was defined as day in vitro (DIV) 0. To investigate the neuronal nature of hiPSC-derived neurons, the expression of *Nestin* (a neural progenitor cell marker), *βIII-tubulin* (a neuron marker), and *MAP2* (a mature neuron marker) was analyzed in hiPSCs at DIV 10, 31, 41, and 51 (Fig. 1b). In early stages of the protocol, cells expressed *Nestin* (DIV 10) and *βIII-tubulin* (DIV 31), suggesting the successful neuronal differentiation of hiPSCs. The gene expression of *Nestin* was decreased from DIV 41 onwards. The levels of gene expression of *MAP2* increased in a time-dependent manner until DIV 41. Extensive immunocytochemical analyses of MAP2 protein in hiPSCs at DIV 33 showed that 54.4 ± 10.3% (mean ± s.e.m., n = 3) of the stained cells were MAP2-positive (Fig. 1c).

**Figure 2:**
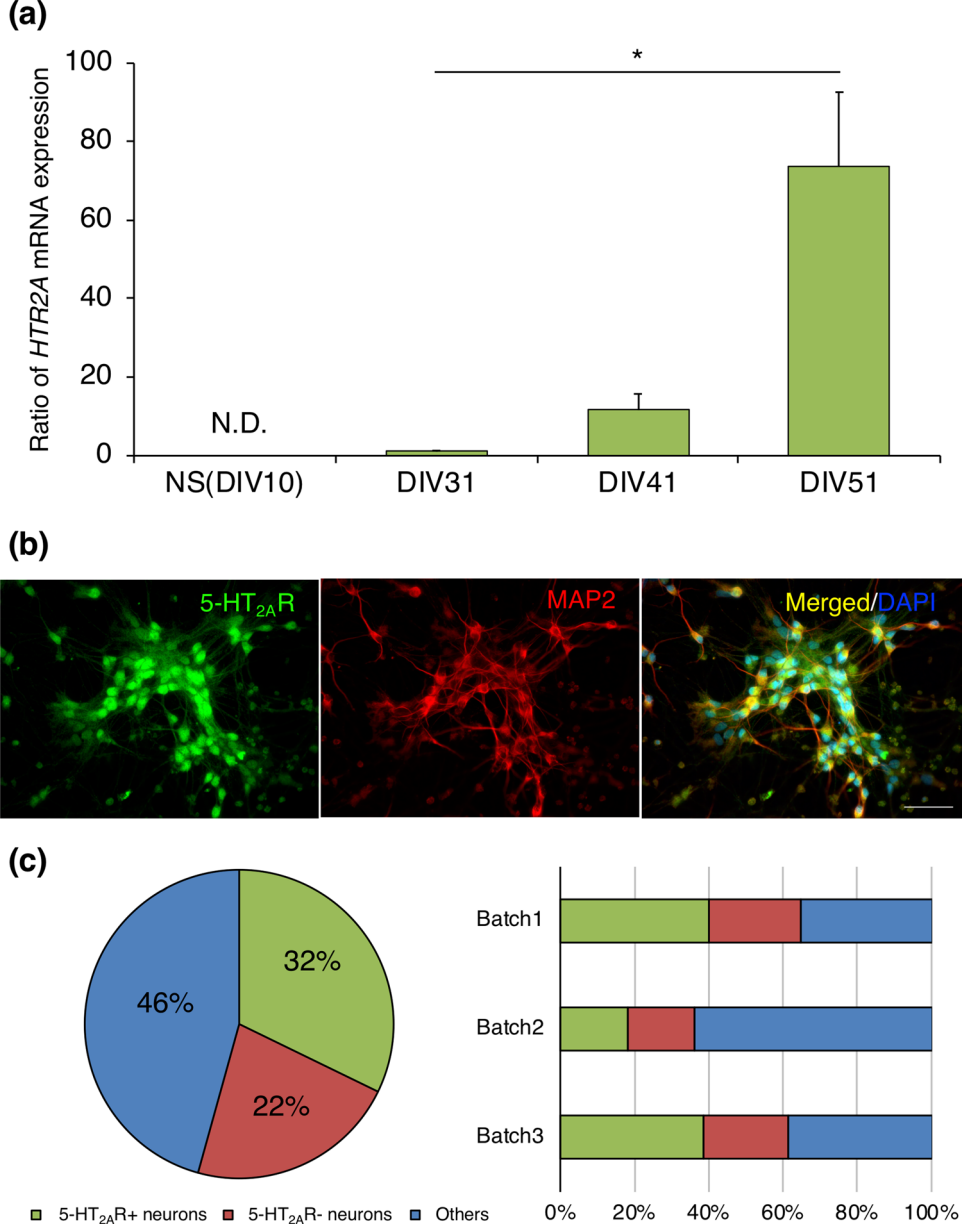
*HTR2A*-positive neurons among induced hiPSCs. (**a**) The gene expression of *HTR2A* in hiPSCs at DIV 10, 31, 41, and 51. The results are expressed as the mean ± s.e.m. of triplicate samples (n = 3) (ANOVA, Dunnet’s test, *p < 0.05). (**b**) Immunofluorescence for 5-HT_2A_R (green), MAP2 (red), and DAPI for nuclear staining (blue). The 5-HT_2A_R- and MAP2-double–positive cells (yellow) are indicated in the merged picture (scale bar = 50 μm). (**c**) Proportion of 5-HT_2A_R-positive and 5-HT_2A_R-negative neurons.

We next investigated the gene expression of *HTR2A*. The expression of *HTR2A* mRNA was first detected after DIV 31. Thereafter, it increased in a time-dependent manner until DIV 51 (Fig. 2a). 5-HT_2A_R protein immunofluorescence was found in 32.2 ± 7.0% of the induced neurons at DIV 33 (Fig. 2b,c).

### Live-cell labelling of hiPSC-derived neurons dependent on *HTR2A* promoter activity

**Figure 3:**
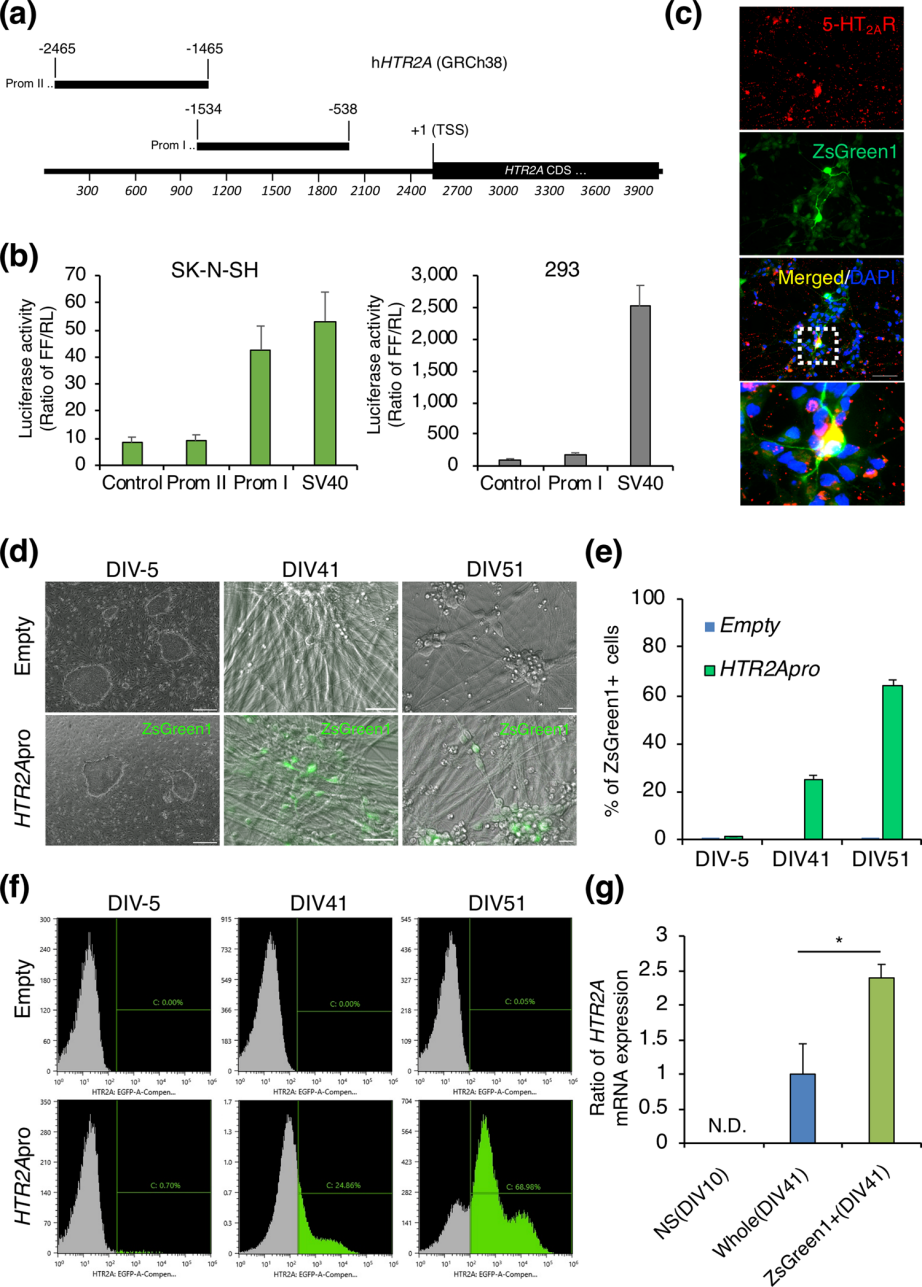
Detection of *HTR2A* promoter-specific fluorescently labelled cells among induced hiPSCs. (**a**) Schematic representation of the *HTR2A* promoter regions: − 1534 to − 538 (Prom I) and − 2465 to − 1465 (Prom II) upstream of the translation start codon (+ 1). Promoter regulatory sequences contained in Prom I. (**b**) Luciferase activity induced by the *HTR2A* promoter in SK-N-SH cells and 293 cells (n = 4). (**c**) 5-HT_2A_R immunofluorescence (red) in hiPSCs at DIV 28 transduced with *HTR2A* promoter-ZsGreen1 (green). A merged image with DAPI for nuclear staining (blue) and extended image are shown at the bottom (scale bar = 50 μm). (**d**) Phase-contrast images of *HTR2A*-promoter specific ZsGreen1 after transduction of the empty vector (top; scale bar = 50 μm) or *HTR2A* promoter-ZsGreen1 (bottom; scale bar = 50 μm) at DIV − 5, 41, and 51. (**e**) Data were analyzed by flow cytometry after transduction of the empty vector (top) or *HTR2A* promoter-ZsGreen1 (bottom) at DIV -5, 41, and 51. The indicated number is the ZsGreen1-expressing cell population. (**f**) ZsGreen1-positive cells as shown by flow cytometry at DIV − 5, 41, and 51. The results are expressed as the mean ± s.e.m. of samples (DIV − 5 and 51: n = 3, DIV 41: n = 5). (**g**) The gene expression of *HTR2A* in whole cells at DIV 10 and 41 and ZsGreen1-positive cells at DIV 41. The results are expressed as the mean ± s.e.m. of samples (NS: n = 3, Whole: n = 4, ZsGreen1^+^: n = 5) (Student’s t-test, N.D. = not detected, *p < 0.01).

To distinguish *HTR2A*-positive neurons from *HTR2A*-negative ones, we generated a lentiviral ZsGreen1-expressing-reporter construct dependent on *HTR2A* promoter activity for the live-cell labelling of neurons (Fig. S1). We amplified two different promoter sequences form human genomic DNA: Prom I and Prom II (Fig. 3a). These sequences were inserted upstream of the luciferase gene of the dual-reporter system. The promoter sequence of Prom I, but not that of Prom II, contains the promoter regulatory sequences ^22^. Human neuroblastoma SK-N-SH cells^23^ were used as *HTR2A*-positive cells, while 293 cells were used as *HTR2A*-negative cells (Fig. S1b). The *HTR2A* promoter activity of the reporter lentiviruses containing Prom I was much higher than that of the reporter containing Prom II in SK-N-SH. However, the *HTR2A* promoter activity of the reporter containing Prom I was not detected in 293 cells (Fig. 3b).

Extensive immunocytochemical analyses of 5-HT_2A_R protein in hiPSCs at DIV 28 transfected with the constructed reporter lentiviruses showed that ZsGreen1-positive cells were co-localized with those that stained positive for 5-HT_2A_R (Fig. 3c). The proportion of 5-HT_2A_R-positive cells among the ZsGreen1-positive cells was 77%, while the proportion of ZsGreen1-positive cells among the 5-HT_2A_R-positive cells was 9% (Fig. S2a–c).

**Figure 4:**
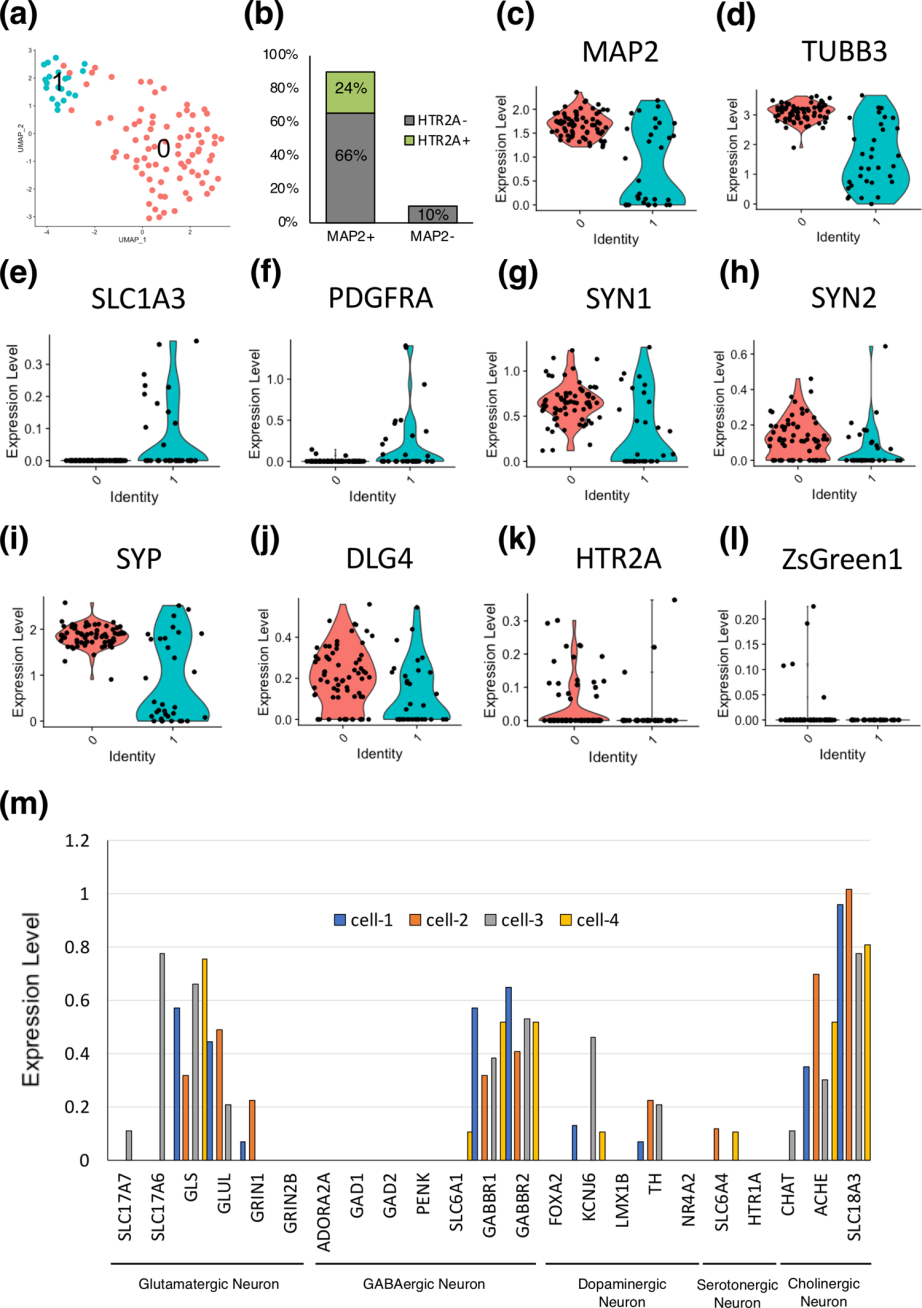
Characterizing the heterogeneity of iPSCs at DIV 51. Single-cell RNA-sequencing of hiPSCs shows neurons and glia. (**a**) UMAP plot of 2 clusters over 102 single cells shows neuronal and glial subtypes. The clusters were determined based on a K-nearest neighbor (KNN) graph. (**b**) Proportion of *MAP2*-positive, *MAP2*-negative, *HTR2A*-positive and *HTR2A*-negative cells. Violin plots of cell subtype markers, (**c**) neurons (*MAP2*), (**d**) immature neurons (*TUBB3*), (**e**) glia (*SLC1A3*), (**f**) oligodendrocyte progenitor cell (*PDGRFA*), (**g**–**j**) mature neurons (*SYN1,SYN2, SYP, DLG4*), and target markers, (**k**) *HTR2A*, (**l**) *ZsGreen1*. Black dots represent the expression level on each cell. (**m**) Gene expression of cell subtype markers in *ZsGreen1* and *HTR2A*-positive cells.

To evaluate the function of the reporter lentivirus, lentivirus-transfected hiPSCs were analyzed using fluorescence microscopy and flow cytometry. The control lentivirus (described as “Empty” in Fig. 3d–f) and constructed reporter lentivirus (described as “*HTR2Apro*” in Fig. 3d–f) were transfected into hiPSCs. The ZsGreen1-positive cells (DIV-5) were not detected by either fluorescence microscopy or flow cytometry (ZsGreen1-positive cells, 0.97 ± 0.17%) (Figs. 2a, 3d–f). The ZsGreen1-positive cells were detected in neurons derived from hiPSCs at DIV 41 and 51 by fluorescence microscopy, and the positive ratio was 25.2 ± 1.5% and 63.9 ± 2.6%, respectively (Fig. 3d–f). Consistent with this, the sorted ZsGreen1-positive cells at DIV 41 showed significantly higher expression of *HTR2A* mRNA than the whole-cell lysate (Fig. 3g). Furthermore, single-cell RNA-sequencing (scRNA-seq) analysis performed with hiPSCs at DIV 51 showed that the induced hiPSCs were derived from neurons and glial lineages, and 90% of these cells showed *MAP2* gene expression (Fig. 4a–f). In addition, these neurons also showed the expression of synaptic markers such as *SYN1*, *SYN2*, *SYP* and *DLG4* (Fig. 4g–j), suggesting that hiPSCs had differentiated into mature neurons. These induced iPSCs-derived neurons showed expression of glutamatergic, GABAergic, cholinergic, dopaminergic, and serotonergic neuronal markers (Fig. S3a–e). A total of 24% of *MAP2*-positive cells were *HTR2A*-positive (Fig. 4b,k). In addition, 5% of the *MAP2*-positive cells were *ZsGreen1*-positive (Fig. 4l), and the *HTR2A*-positive rate was 80% (4 cells/5 cells; Fig. S3f). At the same time, 18% of the *HTR2A*-positive cells were *ZsGreen1*-positive (4 cells/22 cells; Fig. S3g). These results suggested that *ZsGreen1* expression reflects the gene expression of *HTR2A* with high specificity. This is a useful tool to identify *HTR2A*-positive cells among hiPSC-derived neurons. In addition, *HTR2A* and *ZsGreen1*-positive cells showed relatively high expression of glutamatergic, GABAergic and cholinergic neuron markers (Fig. 4m).

### Functional properties of ZsGreen1-positive neurons

To investigate the electrophysiological properties and the responsiveness of *HTR2A*-positive neurons derived from lentivirus-transfected hiPSCs to a 5-HT_2A_R agonist, whole-cell patch-clamp recordings were performed on ZsGreen1-positive (*n* = 15) and negative (*n* = 7) neurons at DIV 42–67.

**Figure 5:**
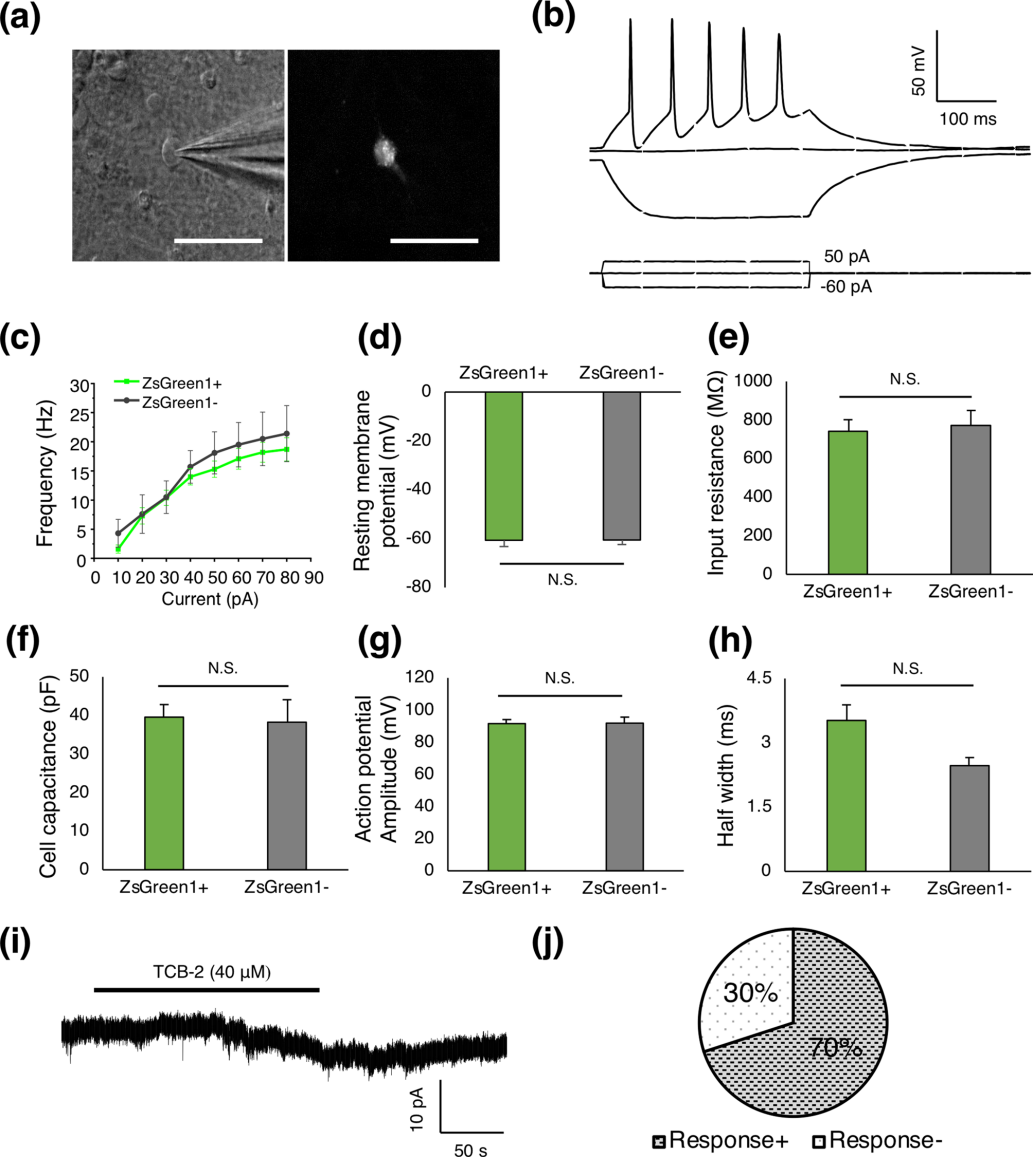
Functional properties of ZsGreen1-positive neurons. (**a**) Translucent (left) and fluorescent (right) images of a ZsGreen1-positive neuron during whole-cell patch-clamp recording at DIV 67 (scale bar = 50 μm). (**b**) Representative traces of membrane potentials in response to injected hyperpolarizing and depolarizing current pulses (− 60, 0, and 50 pA) with 300 ms duration in a ZsGreen1-positive neuron. Repetitive action potentials were evoked by 50-pA injected current. (**c**) Quantitative analysis of frequency-current relationship among ZsGreen1-positive (green) and ZsGreen1-negative (gray) neurons. (**d**–**h**) Quantitative analysis of (**d**) resting membrane potential, (**e**) input resistance, (**f**) cell capacitance, (**g**) action potential amplitude and (**h**) half width of ZsGreen1-positive and ZsGreen1-negative neurons (ZsGreen1-positive: *n* = 15, ZsGreen1-negative: *n* = 7) (Student’s t-test, N.S. = not significant). (**i**) Representative TCB-2-induced current traces in a ZsGreen1-positive neuron. (**j**) Proportion of TCB-2-responsive and nonresponsive neurons (n = 10). Data were analyzed on a personal computer using pCLAMP 10.7 software (Molecular Devices, Sunnyvale, CA, USA, https://www.moleculardevices.com) and Origin 2019 (OriginLab, Northampton, MA, USA, https://www.originlab.com).

In the current-clamp configuration, all recorded cells were capable of generating repetitive action potentials (AP) in response to further depolarizing current injections (Fig. 5a,b). The mean peak AP firing frequency for ZsGreen1-positive and ZsGreen1-negative neurons were 18.7 ± 2.5 Hz and 21.4 ± 4.8 Hz respectively (Fig. 5c). The average resting membrane potential was − 60.7 ± 2.6 mV for ZsGreen1-positive and − 61 ± 1.9 mV for ZsGreen1-negative neurons (Fig. 5d). Input resistance was 741.6 ± 60.2 MΩ for ZsGreen1-positive and 774.0 ± 75.6 MΩ for ZsGreen1-negative neurons (Fig. 5e). Cell capacitance was 39.6 ± 3.3 pF for ZsGreen1-positive and 38.3 ± 5.8 pF for ZsGreen1-negative neurons (Fig. 5f). An Injection of a 300-ms depolarizing current pulse elicited action potentials with amplitude of 91.6 ± 2.5 mV for ZsGreen1-positive and 91.9 ± 3.7 mV for ZsGreen1-negative neurons (Fig. 5g) and half width 3.5 ± 0.4 ms and 2.5 ± 0.2 ms at first spike for ZsGreen1-positive and ZsGreen1-negative neurons, respectively (Fig. 5h).

We next examined the effect of TCB-2, which is a selective agonist of 5-HT_2A_Rs, on the ZsGreen1-positive and negative neurons at a holding potential of − 60 mV under the voltage-clamp configuration. Bath application of TCB-2 (40 μM) produced an inward current (14.1 ± 1.2 pA of amplitude) in 70% of the ZsGreen1-positive neurons (n = 10) (Fig. 5i,j). In contrast, ZsGreen1-negative neurons exhibited no response to TCB-2 (n = 6). These data suggested that ZsGreen1-positive neurons derived from hiPSCs are electrophysiologically active and functionally express 5-HT_2A_R.

## Discussion

The use of hiPSCs derived from patients with a certain neurological disease allows the preparation of brain cells that contain the actual genetic information of the patients themselves^24–27^. This is a notable feature given that such cells have been technologically and ethically difficult to obtain in the past^28^. We have already reported the successful induction of *HTR2A*-expressing neurons of ventral hindbrain region derived from hiPSCs by controlling the regional identity along the anteroposterior axes and the dorsoventral axes^20,29^. In this study, using the same method with slight modifications, we further investigated the time course of the maturation of *HTR2A*-expressing neurons induced from hiPSCs to determine the optimal culture duration for the functional analyses. In these *HTR2A*-expressing neurons, the expression of neuronal markers and the *HTR2A* gene increased over time up to DIV 41 and 51, respectively. Based on this result, we conducted the functional analyses using induced neurons at DIV 41 or later.

To identify the adequate target cells to conduct functional analyses, we developed a reporter lentivirus, which was capable of labelling *HTR2A*. Prom I, the adopted *HTR2A* promoter, showed higher activity than that of Prom II in *HTR2A*-positive cells, but no activity in *HTR2A*-negative cells. This suggested that multiple transcription factors encompassed in Prom I, like Sp1, PEA3, E-box, CRE binding proteins, CACCC box and CCAAT box were interacted^22^. This could be applied not only to electrophysiological analysis of the target cells but also to quantify the proportion of cells expressing *HTR2A* among all the induced cells in the hiPSC culture. The proportion of sorted ZsGreen1-positive cells increased in a time-dependent manner, reaching 64% at DIV 51. This result was consistent with the increase in the expression level of *HTR2A* mRNA. Besides, in scRNA-seq analysis, 80% of the *ZsGreen1*-positive cells were *HTR2A*-positive, which indicated that *ZsGreen1* expression reflects *HTR2A* gene expression with high specificity. We believe that this result is due to the low sensitivity of scRNA-seq. In the flow cytometry experiment, a relatively high percentage (64%) of ZsGreen1-positive cells were MAP2-positive, and we believe that we are able to detect neurons with high sensitivity. In fact, since we used flow cytometry for detection, we expected to be able to detect cells with higher sensitivity than that observed in the scRNA-seq experimental results (18%).

Furthermore, *HTR2A* and *ZsGreen1*-positive cells were likely to be glutamatergic, or GABAergic or cholinergic neurons. Since several neurological diseases might be associated with specific neuronal populations, such as GABAergic neurons^30–33^, it is desirable to develop a tool that can label not only *HTR2A* but also specific types of neurons. Such a strategy should be developed in the future.

Additionally, we successfully showed that *HTR2A*-positive neurons derived from hiPSCs exhibited repetitive action potentials in response to depolarizing current injection, which was consistent with the activity pattern recorded from the neurons induced from hiPSCs in previous studies^34,35^. Regarding the 5-HT_2A_R-specific cell responses, application of TCB-2 generated inward currents in *HTR2A*-positive neurons, which was also consistent with a previous report^36^. These results strongly suggested that the dynamics of the neurons induced from hiPSCs was similar to that of 5-HT_2A_R-expressing neurons in vivo, because 5-HT_2A_R agonists are supposed to cause cell membrane depolarization in these neurons^1,37^.

In conclusion, our in vitro monitoring system allowed the identification and functional analysis of *HTR2A*-positive neurons induced from hiPSCs, which could be used to elucidate the pathophysiological mechanism of various neurological diseases associated with genetic variations of the *HTR2A* gene^7–12,38,39^.

## Methods

All experimental procedures using hiPSCs were approved by the Showa University Ethics Committee for Genome Research (approval no. 179) and the Juntendo University School of Medicine Ethics Committee (approval no. 2016117). The study protocol was in accordance with the 1964 Declaration of Helsinki and its later amendments or comparable ethical standards.

### hiPSC culture

All the experiments were performed with the hiPSC line (C2)^20^. hiPSCs were generated from monocytes in peripheral blood samples of the healthy adult^20^ and formal informed consent was obtained from the subject. hiPSCs were cultured on mitomycin C-treated SNL murine fibroblast feeder cells in standard hESC medium (DMEM/F12, Sigma Aldrich, St. Louis, MO) containing 20% KnockOut Serum Replacement (KSR; Thermo Fisher Scientific, Waltham, MA), 2 mM l-glutamine, 0.1 mM non-essential amino acids (Sigma Aldrich), 0.1 mM 2-mercaptoethanol (Sigma Aldrich), 0.5% penicillin/streptomycin, and 4 ng/ml fibroblast growth factor 2 (FGF-2; PeproTech, Rocky Hill, NJ) in an atmosphere containing 3% CO_2_. The medium was changed every other day.

### Neuronal differentiation of hiPSCs

hiPSCs were pretreated for 5 days with 3 μM SB431542 (Tocris, Bristol, UK), 3 μM dorsomorphin (Sigma Aldrich), and 3 μM CHIR99021 (ReproCELL, Kanagawa, Japan). They were then dissociated and seeded at a density of 10 cells/μl^39^ in KBM Neural Stem Cell medium (Kohjin-Bio, Saitama, Japan) with selected growth factors and inhibitors under conditions of 4% O_2_/5% CO_2_. The growth factors and inhibitors included 1 × B-27 supplement (Thermo Fisher Scientific), 0.5% penicillin/streptomycin, 20 ng/ml FGF-2, 2 μM SB431542, and 3 μM CHIR99021. The neurosphere culture was started at day in vitro (DIV) 0, and neurospheres were passaged at a density of 50 cells/μl on DIV 7 and 14. The following additives were included in the neurosphere culture medium: 10 μM Y-27632 (Fujifilm, Tokyo, Japan) on DIV 0–7 and 100 ng/ml Shh-C24II (R&D Systems, Minneapolis, MN) and 1 μM purmorphamine (Millipore, Burlington, MA) on DIV 1–21. On DIV 21, neurospheres were replated on dishes coated with poly-l-lysine solution and fibronectin and cultured under conditions of 5% CO_2_ at a density of 40 × 10^4^ cells/well in a 48-well plate, 150 × 10^4^ cells/well in a 12-well plate, or 300 × 10^4^ cells/well in a 6-well plate. The medium was changed to Neurobasal Plus Medium (Thermo Fisher Scientific) supplemented with 1 × B-27 Plus supplement (Thermo Fisher Scientific), 0.5% penicillin/streptomycin, 20 ng/ml brain-derived neurotrophic factor (BDNF; BioLegend, San Diego, CA), 20 ng/ml glial cell-derived neurotrophic factor (GDNF; Alomone Labs, Jerusalem BioPark, Israel), 0.2 mM ascorbic acid (Sigma Aldrich), 0.5 mM dbcAMP (Nacalai Tesque, Kyoto, Japan), 1 ng/ml transforming growth factor-β (TGF-β; BioLegend), 10 μM DAPT (Sigma Aldrich), and 3 μM CHIR99021. Half of the volume of medium was replaced with new medium (including all supplements except CHIR99021) every 3 or 4 days.

### Reverse transcription and quantitative polymerase chain reaction (RT-qPCR)

Total RNA was isolated with the RNeasy mini kit (QIAGEN, Hilden, Germany) and the RNeasy micro kit (QIAGEN) with DNase I treatment. cDNA was prepared by using SuperScript First-Standard (Thermo Fisher Scientific) or the TaqMan Fast Advanced Master Mix (Thermo Fisher Scientific). qRT-PCR analysis was performed on a QuantStudio 7 Flex (Thermo Fisher Scientific). Values were normalized to *ACTB* expression. Reactions were carried out in duplicate, and data were analyzed by using the comparative (ddCt) method. Relative expression levels are presented as geometric means ± geometric standard error of the mean (s.e.m.). The primer sets used in the SYBR Green assay are listed in Table 1. Custom primers for the *GAPDH* gene (PN4453320; Thermo Fisher Scientific) and *HTR2A* gene (PN4448892; Thermo Fisher Scientific) were used in the TaqMan assay.

**Table 1:** Primers used for RT-qPCR.

Primer	Primer sequence (5′ → 3′)
Nestin	Forward: TTCCCTCAGCTTTCAGGACCCCAA
	Reverse: AAGGCTGGCACAGGTGTCTCAA
βIII-tubulin	Forward: ATTTCATCTTTGGTCAGAGTGGGC
	Reverse: TGCAGGCAGTCGCAGTTTTCAC
MAP2	Forward: CCGTGTGGACCATGGGGCTG
	Reverse: GTCGTCGGGGTGATGCCACG
Β-actin	Forward: TGAAGTGTGACGTGGACATC
	Reverse: GGAGGAGCAATGATCTTGAT

### Immunofluorescence analysis

Cells were subsequently fixed with 4% paraformaldehyde for 30 min at room temperature and then washed three times with phosphate-buffered solution (PBS). After incubating with blocking buffer (PBS containing 5% normal fetal bovine serum, 0.01% sodium azide, and 0.3% Triton X-100) for 15 min at room temperature, the cells were incubated overnight at 4 °C with primary antibodies diluted with PBS containing 0.05% Triton X-100. As primary antibodies, anti-βIII-tubulin (1:500; Biolegend), anti-5-HT_2A_R (1:200; Millipore), and anti-MAP2 (1:5000; Abcam, Cambridge, UK) antibodies were used. The cells were again washed three times with PBS and incubated with secondary antibodies conjugated with Alexa Fluor 488 (1:500; Thermo Fisher Scientific), Alexa Fluor 594 (1:500; Thermo Fisher Scientific) for 1 h and Hoechst 33342 (1:1000; Sigma Aldrich) for 10 min at room temperature. After washing three times with PBS, samples were mounted on slides and examined by using an all-in-one fluorescence microscope (Keyence, Osaka, Japan).

### Transduction and detection of *HTR2A* promoter activity-induced ZsGreen1 expression in hiPSCs

For lentiviral packaging, the pLV-h*HTR2A*pro-ZsGreen1 plasmid containing the *HTR2A* promoter sequence was co-transfected with the Lentiviral High Titer Packaging Mix (Takara, Shiga, Japan) using Fugene HD (Promega, Madison, WI) into LentiX-293 T cells (Z2180N; Takara). The 48-h–cultured supernatant was filtrated through 0.45-μm pore cellulose acetate filters (Millipore) and concentrated using Centriprep Centrifugal Filters (Millipore). Viral titration was determined by flow cytometric quantification of ZsGreen1-positive cells. Lenti-*HTR2A*-ZsGreen1 particles were transduced into hiPSCs at a multiplicity of infection (MOI) of 10 (Fig. S1). Flow cytometry was performed to measure *HTR2A* promoter activity that induced ZsGreen1 expression in hiPSCs. The lentivirus was transduced into hiPSCs at DIV 22 and hiPSCs were collected at DIV 41 and 51. As a negative control of *HTR2A*, the lentivirus was transduced into hiPSCs at DIV-10 and hiPSCs were collected at DIV-5. ZsGreen1-positive cells were sorted using the SH800 cell sorter (Sony, Tokyo, Japan). Dead cells were excluded by staining with Fixable Viability Dye eFlour 450 (Thermo Fisher Scientific) before fluorescence-activated cell sorting analysis.

### Transfection and luciferase assay

*HTR2A* reporter plasmids were co-transfected with a Renilla luciferase-expressing plasmid (E2241, pRL-TK-luc; Promega) to normalize transfection efficiency in neuroblastoma SK-N-SH cells (RCB0426; RIKEN BRC, Saitama, Japan through the National BioResource Project of the MEXT/AMED, Japan) or 293 cells. Transfection was performed using Fugene HD (Promega) according to the manufacturer’s instructions. pGL4.13-SV40 (E6681; Promega) was used as a positive control. Luciferase activities were measured using the Dual-Glo Luciferase Assay System (E2920; Promega), and the ratio of firefly luminescence to Renilla luminescence was used to express reporter activity.

### Single-cell RNA-sequencing

*HTR2A* transfected hiPSCs at DIV 51 were used for single-cell RNA-seq. The transcriptome library was prepared using Chromium Single Cell 3′ Reagent Kits v3 (10× Genomics) according to the manufacturer’s instructions. The single-cell RNA-seq libraries were sequenced using the DNBSEQ-G400 platforms (MGI Tech Co., Ltd.). Data analysis was performed with Cell Ranger (v4.0.0) and Seurat (v4.0.0).

### Patch-clamp recordings

Whole-cell patch-clamp recordings were performed at room temperature under continuous perfusion with an extracellular solution containing (in mM): NaCl (130), NaHCO_3_ (26), glucose (10), KCl (3), CaCl_2_ (2), MgCl_2_ (2), and NaH_2_PO_4_ (1.25). The solution was oxygenated with 95% O_2_ and 5% CO_2,_ to establish a pH of 7.4. *HTR2A*-positive hiPSCs were identified under fluorescence and infrared differential interference contrast microscopy on an upright microscope (BX51WI; Olympus, Tokyo, Japan) using a 40× water immersion objective, and selected for recordings based on a spherical and bright cell body. ZsGreen1 reporter fluorescence was detected with a U-MNIBA3 filter cube (excitation: 460–495 nm, dichroic mirror: 505 nm, emission: 510–550 nm; Olympus, Tokyo, Japan) and processed with MetaMorph software (MetaMorph; Molecular Devices, Sunnyvale, CA). Patch pipettes were pulled from borosilicate glass (GD-1.5; Narishige, Tokyo, Japan) with a P-97 puller (Sutter, Atlanta, GA). Pipettes were filled with an internal solution containing (in mM): KCl (10), K-gluconate (130), HEPES (10), EGTA (0.4), MgCl_2_ (2), Na_2_-GTP (0.3), and Mg-ATP (2), pH 7.25, 285–300 mOsm. The pipette resistance ranged from 2.5 to 5.0 MΩ when the electrodes were filled with the internal solution. Membrane potentials and currents were recorded with a MultiClamp 700B amplifier and a Digidata 1440A (Molecular Devices) and the data were filtered at 10 kHz, digitized at 20 kHz, and analysed using pCLAMP 10.7 software (Molecular Devices) and Origin 2019 (OriginLab). The measured liquid junction potential of 10 mV was subtracted from all membrane potentials. The cell capacitance and input resistance were calculated under voltage clamp mode from the current to a 10-mV hyperpolarizing step pulse at a holding potential of − 60 mV. TCB-2 induced inward currents were defined as downward deflections of more than 2 standard deviations above baseline.

### Statistical analysis

Statistical analysis was performed with Student’s t-test or one-way analysis of variance (ANOVA) with post hoc Dunnett’s test. The data are presented as the mean ± s.e.m.

## Supplementary Information

Supplementary Information.
