# Supplementary material for: In vitro monitoring of HTR2A-positive neurons derived from human-induced pluripotent stem cells
Source: Sci Rep. 2021 Jul 29;11:15437. doi: 10.1038/s41598-021-95041-3 (PMC8322101; doi:10.1038/s41598-021-95041-3)
Supplement: Supplementary file 1 — Supplementary Information. [file 41598_2021_95041_MOESM1_ESM.docx]

***In vitro* monitoring of *HTR2A*-positive neurons derived from human-induced pluripotent stem cells**

Kento Nakai^1,8^, Takahiro Shiga^2,8^, Rika Yasuhara^3^, Avijite Kumer Sarkar^1^, Yuka Abe^1^, Shiro Nakamura^4^, Yurie Hoashi^1^, Keisuke Kotani^1^, Shoji Tatsumoto^5^, Hiroe Ishikawa^5^, Yasuhiro Go^5,6,7^, Tomio Inoue^4^, Kenji Mishima^3^, Wado Akamatsu^2^, Kazuyoshi Baba^1, *^

^1^Department of Prosthodontics, School of Dentistry, Showa University, 2-1-1 Kitasenzoku, Ohta-ku, Tokyo 145-8515, Japan

^2^Center for Genomic and Regenerative Medicine, School of Medicine, Juntendo University, 2-1-1 Hongo, Bunkyo-ku 113-8421, Japan

^3^Division of Pathology, Department of Oral Diagnostic Sciences, School of Dentistry, Showa University, 1-5-8 Hatanodai, Shinagawa-ku, Tokyo 142-8555, Japan

^4^Department of Oral Physiology, School of Dentistry, Showa University, 1-5-8 Hatanodai, Shinagawa-ku, Tokyo 142-8555, Japan

^5^Exploratory Research Center on Life and Living Systems (ExCELLS), National Institutes of Natural Science, 38 Nishigonaka, Myodaiji, Okazaki-shi, Aichi 444-8585, Japan

^6^Department of System Neuroscience, National Institute for Physiological Science, 38 Nishigonaka, Myodaiji, Okazaki-shi, Aichi 444-8585, Japan

^7^Department of Physiological Science, School of Life Science, SOKENDAI (The Graduate University for Advanced Studies), 38 Nishigonaka, Myodaiji, Okazaki-shi, Aichi 444-8585, Japan

^8^These authors contributed equally

*Correspondence to Kazuyoshi Baba

Phone: +81-3-3787-1151 (Ext.239)

Fax: +81-3-3787-9290

Email: [kazuyoshi@dent.showa-u.ac.jp](mailto:kazuyoshi@dent.showa-u.ac.jp)

**Supplementary Information**

**Supplementary Methods**

**Plasmids.** The sequence upstream of the translation start codon ATG (+1) of human *HTR2A* (NM_000621) was amplified by SpeedSTAR (Takara, Shiga, Japan) using the following primers: 5´-GCTAATAGTTTATCAGAG-3´ and 5´-CCATAGTAATTGGATGTAC-3´ (-1534 to -538 (Prom I)] and 5´-GTGTCCAGAGTGGAATTACTGACA-3´ and 5´-CAATCTTATGAGGAAGGTATTATCCC-3´ (-2465 to -1465 (Prom II)]. Next, the amplified cDNA sequence was subcloned into the pCR2.1-TOPO TA Vector (Thermo Fisher Scientific, Waltham, MA). To measure promoter activity, Prom I and Prom II were cloned into the pGL4 Luciferase Reporter Vector (E6651; Promega, Madison, WI). Several core promoter motifs in the region of -1534 to -538 (Prom I) have been reported previously^1^ (Fig. 3a). A lentiviral expression vector containing the region of -1,534 to -538 (Prom I) and ZsGreen1 (pLV-h*HTR2A*pro-ZsGreen1) was constructed (Fig. S1a; Vector Builder, Chicago, IL). The empty backbone vector without the promoter region was used as a control.

**Supplementary Figure S1**

**
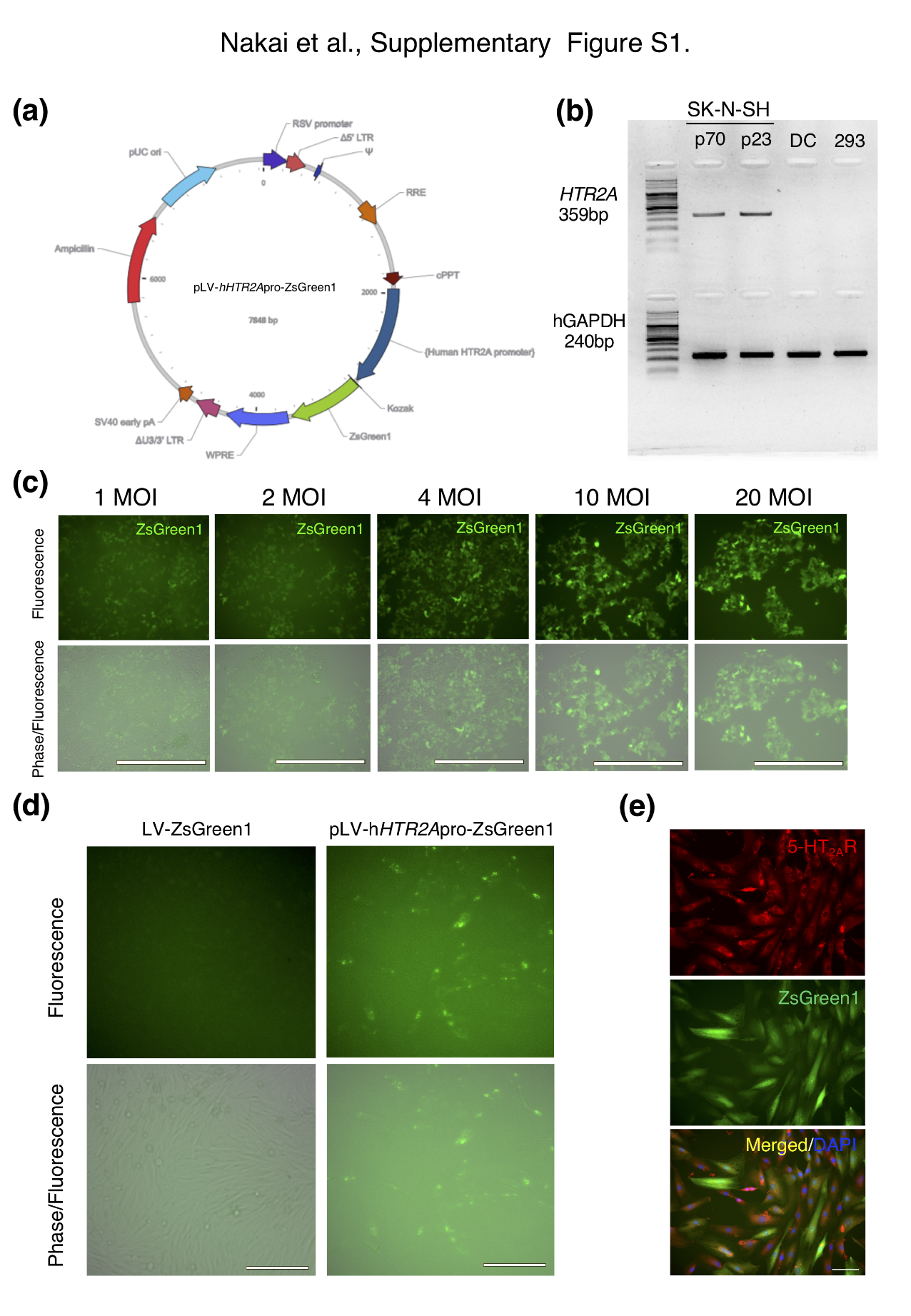
**

**Supplementary Figure S2**

**Supplementary Figure S3**

**Supplementary Figure Legends**

**Supplementary Figure S1. *HTR2A* promoter region design.**

(a) Vector map of the human *HTR2A* promoter–ZsGreen1 lentivector (pLV-h*HTR2A*pro-ZsGreen1). *HTR2A* -Prom I (-1534 to -538) was selected as the target region. (b) The expression of *HTR2A* mRNA was assessed by RT-PCR. Total RNA was isolated from the human neuroblastoma cell line, SK-N-SH, passage 70 (lane 1, p70) and 23 (lane 2, p23); the human salivary ductal cell line, NS-SV-DC (lane 3, DC)^2^; and 293 cells (lane 4). (c) Serial dilutions of lentiviral particles. Lentiviral particles were introduced into 293 cells using the indicated volumes. ZsGreen1 fluorescence was observed under a fluorescence microscope. Merged images of phase-contrast and fluorescence are shown at the bottom (scale bar = 200 μm). (d) Infection of LV-ZsGreen1 (10 multiplicity of infection [MOI]) and pLV-h*HTR2A*pro-ZsGreen1 (10 MOI) into SK-N-SH cells. Merged images of phase-contrast and fluorescence are shown at the bottom (scale bar = 200 μm). (e) 5-HT_2A_R immunofluorescence (red) in SK-N-SH cells transduced with *HTR2A* promoter-ZsGreen1 (green). A merged image with DAPI for nuclear staining (blue) is represented at the bottom (scale bar = 100 μm).

**Supplementary Figure S2. Quantitative analysis of double immunostaining for ZsGreen1 and 5-HT_2A_R at DIV 28.**

(a) The number of ZsGreen1-positive cells and 5-HT_2A_R-positive cells in each batch. (b) The proportion of 5-HT_2A_R-positive cells among the ZsGreen1-positive cells. (c) The proportion of ZsGreen1-positive cells among the 5-HT_2A_R-positive cells.

**Supplementary Figure S3. Characterizing the heterogeneity of iPSCs at DIV 51.**

Violin plots of neuronal cell subtype markers of (a) glutamatergic neuron, (b) GABAergic neuron, (c) dopaminergic neuron, (d) serotonergic neuron, (e) cholinergic neuron. (f) Gene expression levels of *ZsGreen1* and *MAP2* in each analysed cell in scRNA-seq. The square in the scatter plot indicates *ZsGreen1*-positive cells and the proportions of *HTR2A*-positive and *HTR2A*-negative cells. (g) The scatter plot shows the gene expression levels of *ZsGreen1* and *HTR2A* in *HTR2A*-positive cells extracted by scRNA-seq. The proportions of *ZsGreen1*-positive and *ZsGreen1*-negative cells among the *HTR2A*-positive cells were analysed by scRNA-seq.

**Supplementary References**

1. Zhu, Q. S., Chen, K. & Shih, J. C. Characterization of the human 5-HT2A receptor gene promoter. *J. Neurosci.* **15**, 4885–4895 (1995).

2. Azuma, M., Tamatani, T., Kasai, Y. & Sato, M. Immortalization of normal human salivary gland cells with duct-, myoepithelial-, acinar-, or squamous phenotype by transfection with SV40 ori- mutant deoxyribonucleic acid. Lab. Invest. **69**, 24–41 (1993).
